# The Convention Period

**Published:** 1903-07

**Authors:** 


### Editorial.

### THE CONVENTION PERIOD.

The season of recreation is at hand, and the busy practitioner is looking forward with an earnest desire, born of an overstrained body, to that time when, leaving closely confined and not overhealthful rooms, he can enjoy the freedom of air and sunlight.

It is strange that the stomatologist should seek this time of the year to secure rest and to hold his conventions. He apparently accepts this and the torrid heats of an American summer as part of the new invigoration he hopes will be his. He has never been able to answer the question to his satisfaction, Would not some other period be more conducive to health and pleasure? If he has an answer, it will probably be, This is my vacation period, and I must use this or fail to take part in associated effort. June, July, and August are, therefore, the dental convention months and the active mind in dentistry is preparing to take part in State, national, and educational dental conventions.

State conventions, since the origin of dental laws, have assumed, as a rule, much greater prominence than in former years. Those States in which election to the State Boards of Examiners required previous nomination by the State dental societies, have made these bodies the centres of a certain kind of political activity and an added interest to a class of mind not infrequently found in all associations. The usefulness of the State organization was, in former years, an unsolved problem with the writer. Socially it was an event, especially where the meeting was held in some pleasant sea-side or mountain resort, but the scientific value of these meetings has failed to reach a standard commensurate with the amount of labor devoted to them. Why this should have been is difficult to determine, but the explanation may be found in the fact that the number who may have had time and inclination to delve for facts is extremely limited. This being true it is reasonable to suppose that the individual who has spent months in laboratory work would naturally prefer to give forth his conclusions to a larger audience, or, more likely, not give it even there, preferring the more direct contact with a wider range of mind in the dental periodicals of the world, for eventually all productions, good and indifferent, reach this end, the first to live and bear fruit and the last to die.

The conventions of this year are the National Dental Association, which will convene at Asheville, N. C., July 28, and continue four days in session; the National Association of Dental Faculties, which will meet at the same place, Asheville, July 24, and the National Association of Dental Examiners, which will likewise meet at Asheville upon the same date.

The National Association meets for the first time farther south. It was born in the South, at Old Point Comfort, Va., and in its yearly changes to the three points of the compass, East, West, and South, it has, when reaching the latter, gone to the place of its origin. The Northern members anticipate that this change will bring them in contact with a much larger number of their professional colleagues than they usually met with from that section of the United States. While they will miss much of the invigoration of the sea, its loss may be compensated for by the mountain-air of this famed summer resort.

Much interest concentrates about this national body this year, for it must arrange for the International Dental Congress to be held in St. Louis in 1904. That this will be accomplished without friction is not to be expected. Indeed, for a year past the storm centre has changed its location repeatedly between America and Europe. It is a remarkable fact in human nature that, let any movement be inaugurated, whether that be political, religious, philanthropic, or professional, there will be a certain set never quite happy unless they are at the head, or as near to that as possible. This has been true of all international dental congresses held in this country and, possibly, elsewhere. Why men cannot be satisfied to work in the ranks and let the honors of the world fall where they legitimately belong is one of the strange presentations of our complex human nature. There has been war between the committee of *nine* appointed by the International Dental Federation at its meeting in Stockholm, Sweden, to take charge of the Congress, and the committee of *fifteen* appointed by the National Dental Association at Niagara Falls, N. Y., in 1902, for the same service. The contention between these two committees will probably be settled by compromise. At this writing it would seem that the committee of the national body will have the organizing of the Congress. There is much preliminary work to be done in order that this convention may reach the success attained by previous congresses.

The scientific side of the national meeting at Asheville will depend mainly upon the character of the material presented, but, if we may judge from previous gatherings, this will not be an extended advance in the grade of professional work. While this is not to be expected, the good that these national gatherings accomplish is not to be calculated upon this basis, but rather upon the combined effort of widely separated thought upon a single effort. This must always be productive of good through its centralizing power upon the professional mind.

The National Association of Dental Faculties has a continued work to do in maintaining the dental educational standard. It has been a power in the past, and has, through its combined and direct influence, brought dental collegiate instruction in this country up to a standard unthought of a quarter of a century ago, and, if wise counsels continue to prevail, will raise the standard during the twentieth century to a higher plane than is at present possible of attainment. There has been much unjust criticism of this body, that it devotes too much of its time to regulating its membership. This comes from those least familiar with its legitimate business. That it does so regulate is true, but the training of colleges, in membership, and leading them all to a unity of effort, is as much a part of its work as the regulating of the curriculi of the several institutions.

The four years' course, beginning 1903–04, will necessarily claim some attention in this body, but, as that will practically settle itself, it would not seem to require extended thought or discussion.

The National Association of Dental Examiners, of which less is known than any other of the national organizations, as it never publishes its proceedings beyond the list of accepted colleges, would do the dental profession a distinct service if it would take up the interstate relations of dental laws and endeavor to have the restrictions of professional intercourse removed. It is the only organized body that can deal with this somewhat intricate subject.

The time will, in all probability, not soon come when more satisfactory organizations will be formed than those at present existing, and it therefore seems to be the duty of all interested in the development of dentistry to aid to the fullest possible extent in making those we have as valuable as possible, leaving to future generations the duty of building better than we have been able to do with the professional materials within our reach. We are still in the formative period of dentistry, and until that be passed it is hopeless to expect the establishment of a true professional life or work.

